# *Rickettsia monacensis* as Cause of Mediterranean Spotted Fever–like Illness, Italy

**DOI:** 10.3201/eid1804.111583

**Published:** 2012-04

**Authors:** Giordano Madeddu, Fabiola Mancini, Antonello Caddeo, Alessandra Ciervo, Sergio Babudieri, Ivana Maida, Maria Laura Fiori, Giovanni Rezza, Maria Stella Mura

**Affiliations:** University of Sassari, Sassari, Italy (G. Madeddu, A. Caddeo, S. Babudieri, I. Maida, M.L. Fiori, M.S. Mura);; Istituto Superiore di Sanità, Rome, Italy (F. Mancini, A. Ciervo, G. Rezza)

**Keywords:** *Rickettsia monacensis*, MSF–like illness, Sardinia, Italy, Mediterranean spotted fever (Boutonneuse fever), Rickettsia, vector-borne infections

**To the Editor:** *Rickettsia conorii*, the etiologic agent of Mediterrenean spotted fever (MSF), is transmitted to humans by the brown dog tick (*Rhipicephalus sanguineus*). MSF is endemic to Italy; incidence is highest in the south and on the islands of Sardinia and Sicily (*1*). Recently, the use of molecular methods has enabled identification of other rickettsiae of the spotted fever group (SFG) from *Ixodes ricinus* ticks in northeastern Italy and in other areas of Europe (*2**–**6*). *R. monacensis* was identified as an etiologic agent of MSF-like illness in Spain (*7*).

We report a case of MSF-like illness in a 28-year-old man from Sassari in northwestern Sardinia who was admitted to the Infectious Disease Unit of the University of Sassari Hospital in April 2011. At admission, he reported fever (38.2°C) and headache of 2 days’ duration. At physical examination, he had a crusty skin lesion surrounded by edema and erythema, which was compatible with inoculation eschar, on the left calf. He had no rash. Laboratory results showed a slight leukocyte increase, hypocromic and microcytic anemia (hemoglobin 10.6 g/dL [reference range 13.1–17.1 g/dL], mean corpuscular volume 67.7 fL [reference range 81–88 fL], mean corpuscular hemoglobin concentration 29.6 g/dL [reference range 33–35 g/dL]), hyperbilirubinemia (total bilirubin 1.36 mg/dL [reference range 0.2–1.3 mg/dL], direct bilirubin 0.49 mg/dL [reference range 0.0–0.6 mg/dL]), and erythrocyte sedimentation rate 37 mm/h (reference range 0–25 mm/h). The remaining parameters were within reference ranges. A small skin sample taken from the inoculation eschar and whole blood were stored at –30°C. The patient immediately started taking doxycycline 100 mg every 12 hours. Serologic tests were negative for *R. conorii* IgM and IgG (ELISA) and positive for SFG *Rickettsia* spp. IgG on indirect immunofluorescence with a titer of 128. After 24 hours of antimicrobial drug therapy, he was afebrile; he was discharged on day 3. He completed a 7-day course of doxycycline at home and recovered completely.

The skin biopsy sample, collected in phosphate-buffered saline, and whole blood were obtained before antimicrobial therapy began and were subjected to DNA extraction. Bacterial detection and identification were conducted by using molecular methods based on real-time PCR, classical PCR, and nucleotide sequencing (Table).

**Table:** Selected inner primers used to amplify rickettsial *glt*A and *omp*A genes*

Rickettsial groups	Gene	Primer	Nucleotide sequence, 5′ → 3′	Product size, bp	Reference
*Rickettsiae* spotted fever group plus typhus group	*gtlA*	gltA–F	TCGCAAATGTTCACGGTACTTT	74	(*8*)
		gltA–R	TCGTGCATTTCTTTCCATTGTG		
*Rickettsiae ompA*	*ompA*	ompA–F	ATGGCGAATATTTCTCCAAAA	632	(*9*)
		ompA–R	GTTCCGTTAATGGCAGCATCT		

*gltA, citrate synthase; *ompA*, outer membrane protein A.

A set of primers for *glt*A gene that encodes the citrate synthase enzyme (*8*) was used to determine that the organism belonged to the genus *Rickettsia*, which includes the SFG and typhus group. Each real-time PCR reaction was performed by QuantiTect SYBR Green PCR kit (QIAGEN, Hilden, Germany) by using 20 ng of purified DNA. *R. conorii* and *R. typhii* were used as positive controls for SFG and typhus group, and *Anaplasma phagocytophilum*, *Bartonella henselae*, *Ehrlichia chaffeensis*, and *Coxiella burnetii* (*Bartonellaceae* and *Coxiellaceae* members) served as negative controls. Results were checked for the specific molecular length by electrophoresis on a 3% (wt/vol) agarose gel.

The skin biopsy specimen of the inoculation eschar was positive for *Rickettsia* spp. The whole blood sample was negative for *Rickettsia* spp.

These results were confirmed by amplification of the *ompA* gene by using the ompA–F and ompA–R primers (*9*) and by the sequencing of the PCR amplicon. The nucleotide sequence analyzed by using the BLAST search tool (www.ncbi.n/m.nib.gov/blast) showed 100% identity with the *R. monacensis* isolate N72 (GenBank accession no. FJ919650.1). We identified *R. monacensis* as cause of MSF-like illness in the patient reported here.

Our results have several clinical and microbiological implications. Although MSF-like illness is highly endemic to Sardinia, to our knowledge no pathogens other than *R. conorii* had ever been identified. Antibodies against *R. monacensis* were not detected by the *R. conorii* ELISA commonly used in hospital laboratories. In contrast, indirect immunofluorescence, which cannot distinguish between rickettsial species because of cross-reactivity, was positive. Therefore, the cocirculation of *R. monacensis* and, possibly, of other SFG rickettsiae, could lead to misdiagnosis and therapeutic delay. Furthermore, in consideration of the negative result in whole blood, a small skin sample from the eschar might improve the diagnostic sensitivity of PCR.

We did not perform entomologic studies. However, *I. ricinus* ticks, which are considered vectors of *R. monacensis*, are widely distributed in Italy and have been found in Sardinia, although less often than other tick species (*10*). Moreover, it is not excluded that other ticks might act as vectors for *R. monacensis* in Sardinia, where ticks of the genus *Rhipicephalus* are prominent. Molecular investigations of ticks could better clarify the extent of circulation of SFG rickettsiae in Sardinia.

Identification of *R. monacensis* as a cause of MSF-like illness in Sardinia expands the list of pathogenic rickettsiae circulating in Italy. It also highlights the need for further investigation in humans and vectors to understand infection dynamics and improve diagnosis and treatment of this potentially life-threatening disease.
